# *Ex vivo* normothermic preservation of a kidney graft from uncontrolled donation after circulatory death over 73 hours

**DOI:** 10.3389/fbioe.2023.1330043

**Published:** 2024-01-12

**Authors:** Enrique Montagud-Marrahi, Yosu Luque, Ruben Rabadan Ros, Tarek Ajami, Elena Cuadrado-Payan, Hector Estrella, Andres Arancibia, Gerard Sánchez-Etayo, Marc Bohils, Ramsés Marrero, Yilliam Fundora, Maria José Ramírez-Bajo, Elisenda Banon-Maneus, Jordi Rovira, Ana-Belén Larque, Josep Maria Campistol, Fritz Diekmann, Mireia Musquera

**Affiliations:** ^1^ Kidney Transplant Unit. Nephrology and Kidney Transplantation Department. Hospital Clinic of Barcelona, Barcelona, Spain; ^2^ Laboratori Experimental de Nefrologia i Trasplantament (LENIT). Institut d'Investigacions Biomèdiques August Pi i Sunyer (IDIBAPS), Barcelona, Spain; ^3^ Red de Investigación Cooperativa Orientada a Resultados en Salud (RICORS 2040), Madrid, Spain; ^4^ Sorbonne Université - Inserm UMRS_1155, Paris, France; ^5^ Assistance Publique Hopitaux de Paris. Soins Intensifs Nephrologiques et Rein Aigu. Departement de Nephrologie. Hopital Tenon. Paris, France; ^6^ Group of Metabolism and Genetic Regulation of Disease, UCAM HiTech Sport & Health Innovation Hub, Universidad Católica de Murcia, Guadalupe, Spain; ^7^ Kidney Transplant Unit. Urology Department, Hospital Clinic of Barcelona, Barcelona, Spain; ^8^ Donation and Transplant Coordination Section, Hospital Clinic, University of Barcelona, Barcelona, Spain; ^9^ Liver Transplant Unit, Institut Clínic de Malalties Digestives I Metabòliques, Hospital Clinic of Barcelona, Barcelona, Spain; ^10^ Department of Pathology. Hospital Clinic of Barcelona. Corresponding Author: Mireia Musquera, Barcelona, Spain

**Keywords:** normothermic perfusion, kidney transplantation, regenerative medicine, uncontrolled donation, kidney preservation

## Abstract

The transplant community is focused on prolonging the *ex vivo* preservation time of kidney grafts to allow for long-distance kidney graft transportation, assess the viability of marginal grafts, and optimize a platform for the translation of innovative therapeutics to clinical practice, especially those focused on cell and vector delivery to organ conditioning and reprogramming. We describe the first case of feasible preservation of a kidney from a donor after uncontrolled circulatory death over a 73-h period using normothermic perfusion and analyze hemodynamic, biochemical, histological, and transcriptomic parameters for inflammation and kidney injury. The mean pressure and flow values were 71.24 ± 9.62 mmHg and 99.65 ± 18.54 mL/min, respectively. The temperature range was 36.7°C–37.2°C. The renal resistance index was 0.75 ± 0.15 mmHg/mL/min. The mean pH was 7.29 ± 0.15. The lactate concentration peak increased until 213 mg/dL at 6 h, reaching normal values after 34 h of perfusion (8.92 mg/dL). The total urine output at the end of perfusion was 1.185 mL. Histological analysis revealed no significant increase in acute tubular necrosis (ATN) severity as perfusion progressed. The expression of KIM-1, VEGF, and TGFβ decreased after 6–18 h of perfusion until 60 h in which the expression of these genes increased again together with the expression of *β*-catenin, Ki67, and TIMP1. We show that normothermic perfusion can maintain a kidney graft viable *ex vivo* for 3 days, thus allowing a rapid translation of pre-clinical therapeutics to clinical practice.

## Introduction

*Ex vivo* kidney graft preservation strategies have become a promising strategy to minimize ischemia–reperfusion injury with the aim of reducing the risks of primary non-function and post-transplant delayed graft function (DGF) (ML and SA, 2013; Hamar and Selzner, 2018; Kataria et al., 2019; Rijkse et al., 2021). Hypothermic machine perfusion (HMP) has been demonstrated to improve organ preservation compared to static cold storage (SCS) and is currently widely used for kidney graft preservation (Summers et al., 2015; Tedesco-Silva et al., 2017; Kataria et al., 2019; Tingle et al., 2019). However, HMP allows a limited evaluation of graft quality during the preservation period, given that the organ is not in a physiological setting during HMP (8). The normothermic machine perfusion (NMP) system has been developed to provide a more precise metabolic and functional evaluation of the kidney graft and is one of the most promising strategies for kidney graft *ex vivo* preservation (Chandak et al., 2019; Hamelink et al., 2022; Mazilescu et al., 2022). Unlike hypothermic perfusion, NMP provides physiological conditions with full metabolic and tissue repair activity (Hosgood et al., 2015; Elliott et al., 2020). NMP raises the possibility of maintaining the graft under conditions suitable for transplantation even when the *ex vivo* time is prolonged (Kaths et al., 2018; Hosgood et al., 2023). At present, one of the most important efforts of the kidney transplant community is focused on prolonging the *ex vivo* preservation time of kidney grafts, not only to allow for long-distance kidney graft transportation but also to assess the viability of marginal kidney grafts (Kaths et al., 2018). Furthermore, long-term normothermic perfusion can transform NMP into a valuable system for the translation of innovative medicine strategies to clinical practice, especially those focused on cell and vector delivery to organ conditioning and reprogramming (Gregorini et al., 2017; Thompson et al., 2021; Mellati et al., 2022; Thompson et al., 2022).

Here, we describe the first case of successfully preserving a kidney from a donor after uncontrolled donation after circulatory death (uDCD) over a 73-h period using NMP. We analyze the perfusion and hemodynamic parameters during this preservation time as well as kidney allograft histology.

## Materials and methods

### Patients

A single-center study was performed in the Hospital Clinic of Barcelona on one discarded human kidney for transplantation. The patient was declared as a potential donor, and the organs were evaluated according to our center policy. Only after the decision to be discarded according to clinical criteria, the kidney was accepted for research purposes. The study protocol was reviewed and approved by the Research Ethics Committee of our center (Comité de Ética de la Investigación con medicamentos, CEIm). Written informed consent to participate in this study was provided by the patient’s relatives.

### Normothermic perfusion setup and sample collection

Normothermic Perfusion was performed using the ARK Kidney device from EBERS Medical^®^, and urine recirculation was not performed. The renal artery was cannulated to the circuit using a 12-Fr cannula; renal vein outflow was collected via a pump to an oxygenator, whereby blood re-entered the main circuit. The ureter was directly cannulated to the urine circuit, and urine was collected without recirculation. A urine flow sensor monitored the urine output. Instead of urine recirculation, repositioning with a balanced solution (Isofundin^®^) according to the urine output was conducted via a pump connected to the urine flow sensor. In addition to the urine outflow, the ARK Kidney device continuously monitors hemoglobin concentration, oxygen saturation, mean pressure, arterial flow, renal resistance index, and perfusate temperature. Hemodynamics were pressure-controlled, establishing a pressure increase ramp until reaching a set mean arterial pressure of 70 mmHg during perfusion. The temperature was set at 37°C during perfusion.

The system was primed with two units of packed isogroup red blood cells (RBCs) and 500 mL of balanced saline solution (Isofundin^®^), with an approximate total volume of 800 mL. Oxygenation of the perfusate was performed by manual regulation of air (21% oxygen) at a flow rate of 1.5 mL/min to maintain an oxygen saturation level of over 97% using an ECMO oxygenator. A nutrition solution was continuously infused at 24 mL/h. The nutrition solution was composed of 10% Aminoplasmal^®^, Cernevit^®^ as a multivitamin complex, 5% serum glucose, and 70 UI of insulin. Verapamil was used as a vasodilator (continuous infusion rate, 0.4 mg/h). Before kidney connection to the circuit, 1.2 g of amoxicillin clavulanate, 10 mL of sodium bicarbonate 8.4%, 8 mg of dexamethasone, 2,000 UI of low-molecular-weight heparin, 5 mL of glucose 5%, and 0.5 mL of calcium gluconate were added. The same dose of antibiotic was administered every 24 h. A measure of 10 mL of sodium bicarbonate 8.4% was administered as required to maintain a pH > 7.30. Finally, 5 mL of glucose 5% was administered as required to maintain a perfusate glucose >100 mg/dL.

Biochemical parameters were measured in the perfusate using the epoc^®^ Blood Analysis System (Siemens Healthcare). Perfusate samples were collected hourly during the first 6 h and every 4 h afterward. Histological samples were collected 15 min after starting the perfusion (time point 0) and at 6, 18, 24, 36, 48, 60 and 72 h. A 1 × 1 × 1 mm tissue piece of the kidney cortical was taken from the middle third of the graft and immediately preserved in RNAprotect^®^ (Qiagen) for 24 h. Afterward, the sample was preserved at −80°C until analysis. In case of bleeding after the biopsy, the tissue was carefully sutured to avoid leakage and ensure that renal flow was not affected.

To assess acute tubular necrosis (ATN) severity in the tissue samples, a 5-grade classification according to the frequency of ATN findings in the kidney cortex was performed (Tavares et al., 2012): ATN grade 0 (normal), ATN grade 1 (only rare tubules with evidence of necrosis are observed in the cortex), ATN grade 2 (small groups of necrotic tubules discontinuously distributed throughout the renal cortex), ATN grade 3 (groups of necrotic tubules are easily found in the renal cortex), and ATN grade 4 (extensive areas of tubular necrosis are scattered throughout the renal cortex).

### Real-time qPCR

The kidney tissue was homogenized, and total RNA was extracted using the Maxwell^®^ RSC Instrument (Promega). The Maxwell^®^ RSC miRNA Tissue Kit (Promega) was used, according to the supplier’s protocol.

cDNA was synthesized from the RNA template using a cDNA synthesis kit from Invitrogen, according to the manufacturer’s instructions. The resulting cDNA was diluted and used to determine expression levels. The reference gene used was human *β*-actin, and the expression levels of kidney injury molecule 1 (KIM-1), vascular endothelial growth factor (VEGF), transforming growth factor *β* (TGFβ), tissue inhibitor of metallopeptidase 1 (TIMP1), *β*-catenin, and Ki67 were measured.

Real-time qPCR was performed using the corresponding primers for each gene on a 384-well plate using the PCR program provided by the supplier on a QuantStudio 7 device (Thermo Fisher Scientific). Samples were run in triplicate in 10 μL reaction volumes, and the mRNA expression of the target genes was normalized to *β*-actin mRNA and expressed as the fold change to time 0 (T0) kidney tissue using the ΔΔCT method.

### Statistical analysis

Data are presented as mean (standard deviation, SD). For real-time qPCR, the mRNA expression of the target genes was normalized to *β*-actin mRNA and expressed as the fold change to time 0 (T0) kidney tissue using the ΔΔCT method. Graphical representation was conducted using GraphPad v.9 (GraphPad Software, La Jolla, CA, US).

## Results

The donor was a 55-year-old man with no past medical history who experienced a cardiac arrest due to a pulmonary embolism. Cardiopulmonary resuscitation (CPR) was initiated immediately, and the patient was transferred to the hospital. CPR was unsuccessful after 25 min, and the patient was declared a potential organ donor. In line with our center’s uDCD policy, an *in situ* regional normothermic perfusion for abdominal organs was performed until kidney retrieval in the operating room. The functional warm ischemia time was 30 min, and normothermic regional perfusion was performed for 1.5 h until kidney retrieval. Abdominal cannulation and normothermic regional perfusion were performed as previously published (Hessheimer et al., 2022). The serum creatinine level immediately before donation was 1.67 mg/dL, likely due to acute kidney injury (AKI). No preimplantation biopsy was performed, and no other clinical, histological, or laboratory features that contraindicate donation were evidenced. After kidney retrieval, a malignant neoplasm was identified in the right kidney, and thus both kidneys were discarded for transplantation. The left kidney was used for research purposes. The left kidney was first flushed and then subsequently immersed in IGL-1 cold preservation solution and stored on ice at 4°C until NMP. The total cold ischemia time was 12 h.

**FIGURE 1:**
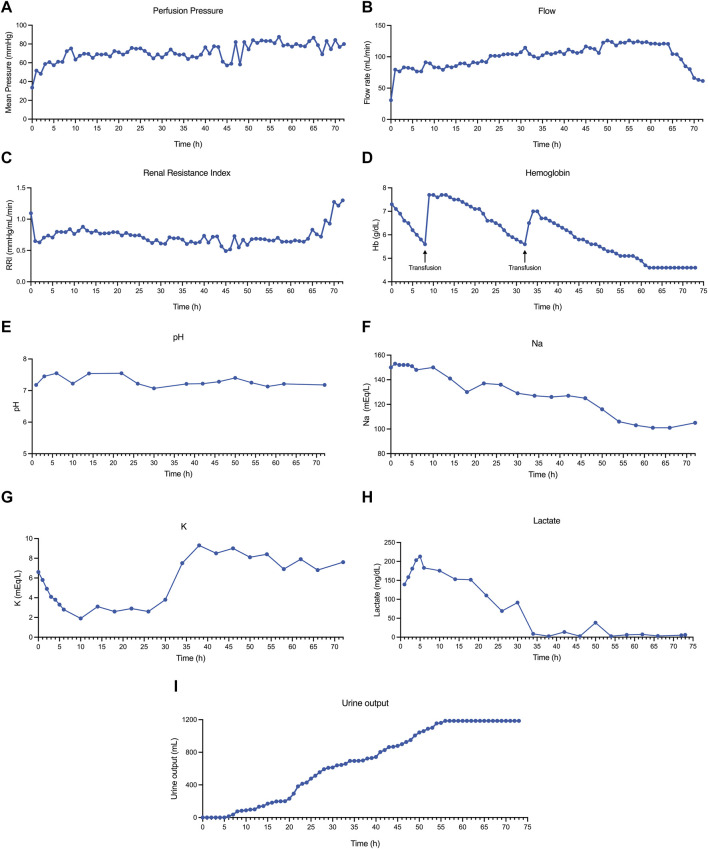
Hemodynamic and biochemical analyses during kidney perfusion. **(A)** Mean perfusion pressure. **(B)** Flow rate during perfusion. **(C)** Renal resistance index (RRI) during perfusion. **(D)** Perfusate hemoglobin during perfusion. The black arrows represent red blood cell transfusions. **(E)** Perfusate pH during kidney perfusion. **(F)** Perfusate sodium during perfusion. **(G)** Perfusate potassium during perfusion. **(H)** Lactate levels during perfusion. **(I)** Kidney urine output.

Hemodynamics were pressure-controlled, establishing a pressure increase ramp until reaching a set mean arterial pressure of 70 mmHg during perfusion. To avoid excessive pressure and parenchyma damage, the pressure was set at 30 mmHg during the first hour and increased by 10 mmHg/h until a pressure of 60 mmHg. When the organ reached 6 h of perfusion, the pressure was increased to 70 mmHg (mean 71.24 ± 9.62 mmHg). The mean flow values were 99.65 ± 18.54 mL/min. The temperature was set at 37°C during perfusion (range 36.7°C–37.2°C). Figures 1A, B show the progression of pressure and flow values during perfusion. The renal resistance index (RRI) fell dramatically after the first hour of perfusion from 1.09 to 0.65 mmHg/mL/min, with a mean value of 0.75 ± 0.15 mmHg/mL/min (Figure 1C). After 65 h of perfusion, an important increase in the RRI was observed from 0.83 to 1.30 mmHg/mL/min, which was accompanied by a decrease in arterial flow (Figure 1C).

**FIGURE 2:**
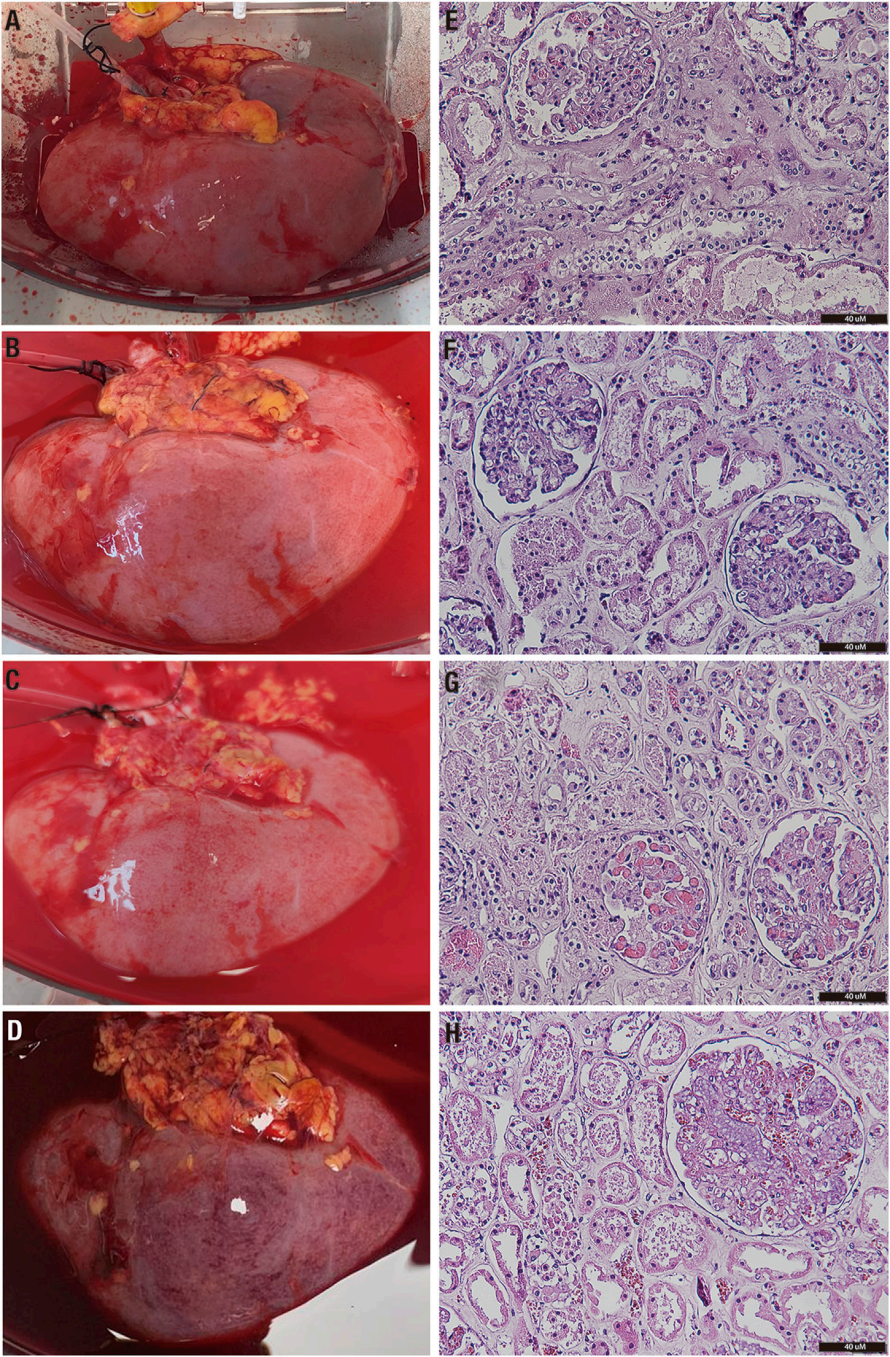
Macro- and microscopical assessment of the kidney graft during perfusion. **(A)** Macroscopical assessment before perfusion onset (time 0). **(B)** Macroscopical assessment at 24 h of perfusion. **(C)** Macroscopical assessment at 48 h of perfusion. **(D)** Macroscopical assessment at 73 h of perfusion. **(E)** H&E staining of a kidney biopsy at time 0 (optical microscope, ×20). **(F)** H&E staining of a kidney biopsy at 24 h (optical microscope, ×20). **(G)** H&E staining of a kidney biopsy at 48 h (optical microscope, ×20). **(H)** H&E staining of a kidney biopsy at 72 h (optical microscope, ×20). H&E, hematoxylin–eosin.

Perfusate hemoglobin was maintained at >5.5 g/dL during perfusion, infusing 1 unit of a packed isogroup red blood cells (RBCs) when the hemoglobin dropped below this value. In total, 3 units of RBCs were administered during 73 h of perfusion, and 2 of them were administered simultaneously at 32 h of perfusion (Figure 1D). The mean pH was 7.29 ± 0.15 (Figure 1E), and a total of 50 mL of 8.4% bicarbonate was added during all the perfusion procedures to maintain the pH value. Perfusate sodium experienced a progressive decay, which became more pronounced after 50 h of perfusion (Figure 1F). In contrast, normal (or even lower) levels of potassium were evidenced until 40 h of perfusion, in which a marked hyperkalemia level was observed (Figure 1G). The lactate concentration increased during the first 6 h (peak of 213 mg/dL). Following this, the lactate concentration started to fall, reaching normal values after 34 h of perfusion (nadir 2.7, normal <20 mg/dL) (Figure 1H). This improvement in lactate concentration was accompanied by an increase in the urine output (Figure 1I); the total urine output at the end of perfusion was 1.185 mL. A significant improvement in the macroscopic aspect of the kidney (from pale to uniform light pink color) was observed from 24 h of perfusion and was maintained until 70 h of perfusion. Following this time, the kidney deteriorated macroscopically, and perfusion was terminated at 73 h (Figures 2A–D).

**FIGURE 3:**
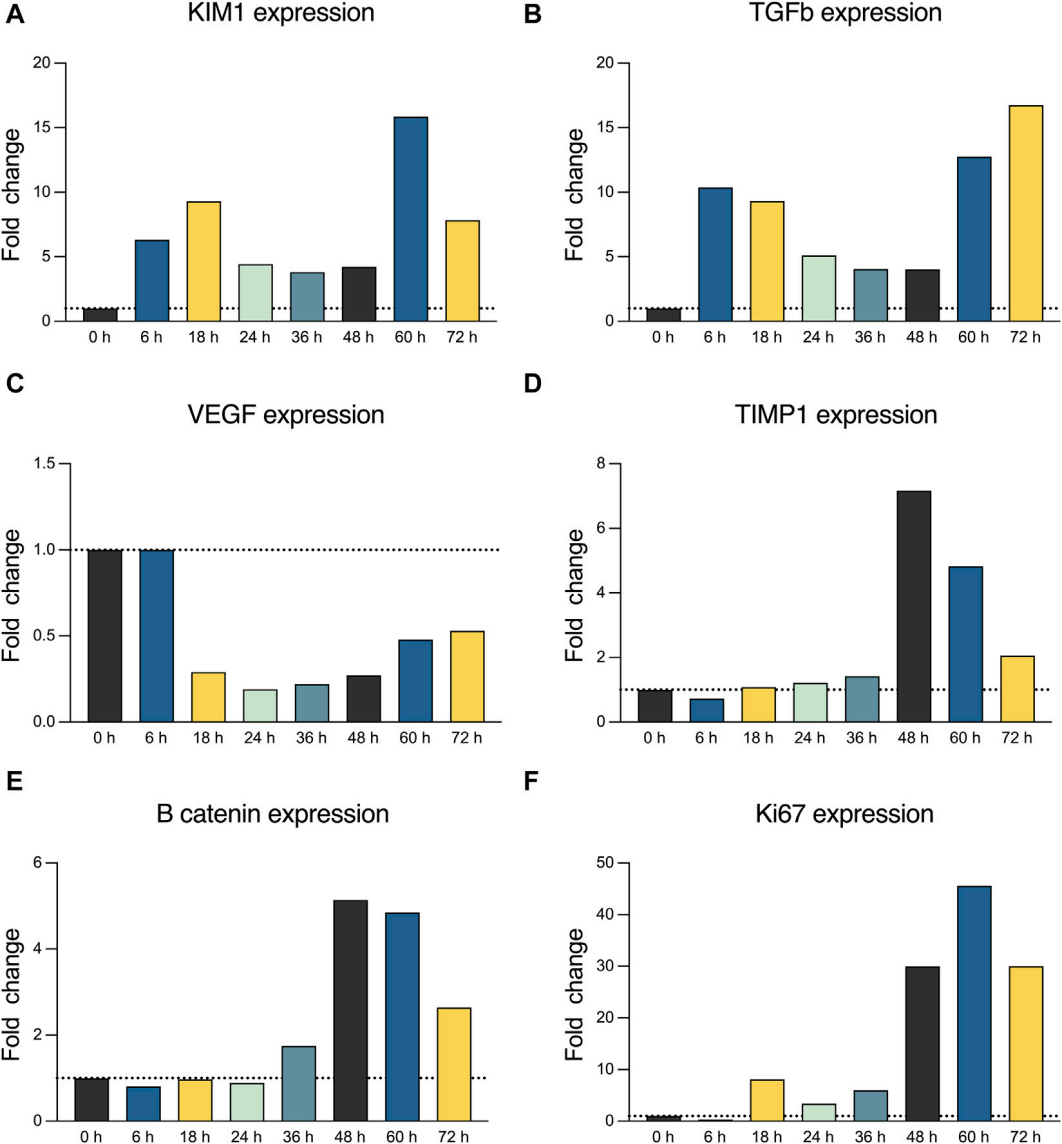
**(A)** Real-time qPCR analysis for KIM-1 expression. **(B)** Real-time qPCR analysis for TGFβ expression. **(C)** Real-time qPCR analysis for VEGF expression. **(D)** Real-time qPCR analysis for TIMP1 expression. **(E)** Real-time qPCR analysis for *β*-catenin expression. **(F)** Real-time qPCR analysis for Ki67 expression.

Histological analysis revealed only a few areas, suggesting an ATN grade 2 in the collected samples. Notably, no significant increase in ATN severity was observed as perfusion progressed, with preservation of the tubular and interstitial compartments when compared to the kidney before perfusion. Nevertheless, significant glomerular congestion was identified at 48 and 72 h. Only one glomerulus was sclerosed in the samples analyzed (Figures 2E–H). We further analyzed the expression of AKI (KIM-1), inflammation (VEGF and TGFβ), and proliferation and repair (Ki67, TIMP1, and *β*-catenin) markers to assess tissue response to long-term normothermic perfusion. Globally, we observed a reduction in KIM-1, VEGF, and TGFβ expression after 6–18 h of perfusion until 60 h, in which the expression of these genes increased again together with the expression of *β*-catenin, Ki67, and TIMP1 (Figure 3).

## Discussion

With this report, we describe the first case of feasible 73-h preservation of an uDCD kidney using normothermic perfusion. This was associated with an increasing urine output over time, an improvement in organ ischemia, and alongside histological and transcriptomic signs of organ viability.

Beyond the advantages regarding the physiological preservation of the kidney graft and the possibility of assessing kidney viability, NMP creates a time window for therapeutic interventions, especially when the administration and analysis *in vivo* are complex and need a physiological environment (Hosgood et al., 2015; Gregorini et al., 2017; Kaths et al., 2018; Hamelink et al., 2022; Mellati et al., 2022). Nevertheless, most of the current regeneration strategies need a relatively long organ preservation time to observe their effects (Gregorini et al., 2017). In 2018, Weissenbacher et al. (2019) perfused 13 discarded human kidneys for 24 h, showing a slight improvement in histological parameters, although KIM-1, as an AKI biomarker, did not show a significant decrease over time, and lactate levels at 24 h were higher than those found in our study. The same group later reported an extended successful perfusion of a DBD discarded kidney graft for 48 h, with lower lactate levels and preserved tubular integrity over time (Weissenbacher et al., 2022). In contrast, we achieve, for the first time, a preservation time of 73 h of an ECD and uDCD kidney graft, thus suggesting that long-term normothermic preservation of ECD kidneys for transplantation is feasible, and its potential utilization is beyond kidney preservation for transplantation, since it can be used as a bioreactor to assess innovative treatment strategies in a specific and safe manner, which usually requires a relatively long time to produce any effect in the organ (Sampaziotis et al., 2021; Thompson et al., 2021; Mellati et al., 2022; Thompson et al., 2022).

Among the different parameters we assessed, we observed a fall in lactate concentration to normal values, which suggests a restoration of kidney metabolism during NMP. This finding contrasts with those reported by Weissenbacher et al., in which lactate levels did not fall under 60 mg/dL, which can be related to the longer perfusion period performed in our study and the longer time the kidney needs for restabilizing its metabolic activity (Weissenbacher et al., 2019; Weissenbacher et al., 2022). This was accompanied by macroscopic improvement and an increasing urine output as a sign of functional recovery, similar to that previously reported for shorter perfusions (Weissenbacher et al., 2019; Weissenbacher et al., 2022). Finally, we assessed the transcriptomic print through qPCR analysis, which suggested a reduction in the initial AKI and inflammation response over time until 60 h of perfusion. These findings are also in line with those reported by Weissenbacher et al., although, in that case, KIM-1 was measured in the perfusate and no significant decrease was observed (Weissenbacher et al., 2019). At 60 h, kidney damage and inflammation increased again until 73 h, at which point perfusion was terminated due to a significant worsening of the macroscopic appearance of the kidney. The increased expression of TIMP1, TGFβ, VEGF, and *β*-catenin after this time point reinforced an enhancement of the inflammation–fibrosis pathway after an AKI, especially after a hypoxia-related injury (Cai et al., 2008; Gu et al., 2020; Miao et al., 2022). This worsening was accompanied by a rapid increase in RRI and flow, thus suggesting a case of progressive kidney graft failure. We hypothesize that these findings were related to the lower levels of hemoglobin because hemolysis, after 60 h, can cause an intrinsic complication of long *ex vivo* perfusions that can compromise tissue integrity, thus preventing the long-term maintenance of the organ (Hosgood et al., 2022). The extent of this hemolysis is further represented by the increase in perfusate potassium, which occurs after the third red blood cell transfusion and the subsequent accelerated decay in hemoglobin. To overcome this obstacle, the maintenance of higher hemoglobin levels (together with periodical perfusate replacement) or the use of non-hemoglobin oxygen carriers can be useful to mitigate hemolysis of long-term normothermic perfusions (Laing et al., 2017; Aburawi et al., 2019; Bhattacharjee et al., 2020). Noticeably, a decrease in sodium perfusate was evidenced, especially after 60 h. This decay in sodium levels can be related to the absence of urine recirculation, which has been suggested to improve hydroelectrolytic balance during kidney perfusion (Weissenbacher et al., 2021). The absence of these approaches, together with progressive fluid administration through crystalloids and blood, can justify the occurrence of hyponatremia.

Our study has some limitations. First, it is a single-center study and a single-case report, and its conclusions have to be confirmed in further studies. Second, urine composition and electrolyte gradients between perfusate and urine could not be addressed since the system used for biochemical assessment is not able to analyze urine. Third, the perfused kidney was not transplanted in a human, although one of the main aims of the present study was to propose normothermic perfusion as a promising platform for long-term organ preservation in order to test innovative therapies and translate them to clinics. Nevertheless, and despite our preliminary results, our report shows the first case of an ECD and uDCD human kidney graft perfused more than 48 h, and it reinforces the idea of NMP as a promising platform for long-term solid organ preservation and drug testing in human organs in a specific and safe manner.

In conclusion, we have shown that NMP could recover biological parameters and urine output in a kidney from uDCD with ischemic AKI and that the organ can be maintained viable *ex vivo* for 3 days. These results suggest that NMP, together with improving strategies for long-term perfusion of solid organs, may permit a rapid translation of pre-clinical approaches to clinical practice.

## Data Availability

The raw data supporting the conclusion of this article will be made available by the authors without undue reservation.

## Abbreviations

AKI, acute kidney injury; ATN, acute tubular necrosis; CPR, cardiopulmonary resuscitation; DGF, delayed graft function; H&E, hematoxylin–eosin; HMP, hypothermic machine perfusion; KIM-1, kidney injury molecule 1; NMP, normothermic machine perfusion; RBCs, red blood cells; RRI, renal resistance index; SCS, static cold storage; uDCD, donor after uncontrolled circulatory death; TGFβ, transforming growth factor *β*; VEGF, vascular endothelial growth factor; TIMP1, tissue inhibitor of metallopeptidase 1.
