# Corrigendum: *Ex vivo* normothermic preservation of a kidney graft from uncontrolled donation after circulatory death over 73 hours

**DOI:** 10.3389/fbioe.2024.1397979

**Published:** 2024-03-21

**Authors:** Enrique Montagud-Marrahi, Yosu Luque, Ruben Rabadan Ros, Tarek Ajami, Elena Cuadrado-Payan, Hector Estrella, Andres Arancibia, Gerard Sánchez-Etayo, Marc Bohils, Ramsés Marrero, Yilliam Fundora, Maria José Ramírez-Bajo, Elisenda Banon-Maneus, Jordi Rovira, Ana-Belén Larque, Josep Maria Campistol, Fritz Diekmann, Mireia Musquera

**Affiliations:** ^1^ Kidney Transplant Unit, Nephrology and Kidney Transplantation Department, Hospital Clinic of Barcelona, Barcelona, Spain; ^2^ Laboratori Experimental de Nefrologia i Trasplantament (LENIT), Institut d’Investigacions Biomèdiques August Pi i Sunyer (IDIBAPS), Barcelona, Spain; ^3^ Red de Investigación Cooperativa Orientada a Resultados en Salud (RICORS 2040), Madrid, Spain; ^4^ Sorbonne Université–Inserm UMRS_1155, Paris, France; ^5^ Assistance Publique Hopitaux de Paris, Soins Intensifs Nephrologiques et Rein Aigu, Departement de Nephrologie, Hopital Tenon, Paris, France; ^6^ Group of Metabolism and Genetic Regulation of Disease, UCAM HiTech Sport & Health Innovation Hub, Universidad Católica de Murcia, Guadalupe, Spain; ^7^ Kidney Transplant Unit, Urology Department, Hospital Clinic of Barcelona, Barcelona, Spain; ^8^ Donation and Transplant Coordination Section, Hospital Clinic, University of Barcelona, Barcelona, Spain; ^9^ Liver Transplant Unit, Institut Clínic de Malalties Digestives I Metabòliques, Hospital Clinic of Barcelona, Barcelona, Spain; ^10^ Department of Pathology, Hospital Clinic of Barcelona, Barcelona, Spain

**Keywords:** normothermic perfusion, kidney transplantation, regenerative medicine, uncontrolled donation, kidney preservation

In the published article, there was an error in the **Funding** statement. The code of the Instituto Carlos III project was incorrectly written as “PI17/00108”. It should instead be “PI21/00205”. The correct Funding statement appears below.

